# Monoplacophoran mitochondrial genomes: convergent gene arrangements and little phylogenetic signal

**DOI:** 10.1186/s12862-016-0829-3

**Published:** 2016-12-16

**Authors:** I. Stöger, K. M. Kocot, A. J. Poustka, N. G. Wilson, D. Ivanov, K. M. Halanych, M. Schrödl

**Affiliations:** 1 SNSB-Bavarian State Collection of Zoology, Muenchhausenstrasse 21, 81247 Munich, Germany; 2 Department of Biological Sciences, University of Alabama, Box 870344, Tuscaloosa, AL 35487 USA; 3 Max-Planck Institut fuer Molekulare Genetik, Evolution and Development Group, Ihnestrasse 73, 14195 Berlin, Germany; 4 Dahlem Center for Genome Research and Medical Systems Biology, Environmental and Phylogenomics Group, Fabeckstraße 60-62, 14195 Berlin, Germany; 5 Alacris Theranostics GmbH, Fabeckstr. 60-62, 14195 Berlin, Germany; 6 Western Australian Museum, Aquatic Zoology/Molecular Systematics Unit, 49 Kew Street, Welshpool, WA 6106 Australia; 7 Zoological Museum, Moscow State University, Bolshaya Nikitskaya Str. 6, 225009 Moscow, Russia; 8 Biological Sciences Department, Auburn University, Life Sciences Bld. 101, Auburn, AL 36849 USA; 9 Faculty of Biology, Department II, Ludwig-Maximilians-Universitaet Muenchen, Großhaderner Strasse 2-4, 82152 Planegg-Martinsried, Germany; 10 GeoBio-Center at LMU, Richard-Wagner-Strasse 10, 80333 Munich, Germany

**Keywords:** Mollusca, Mitogenome, Monoplacophora, Serialia, Aculifera, Conchifera, Gene arrangement, Phylogeny, Evolution

## Abstract

**Background:**

Although recent studies have greatly advanced understanding of deep molluscan phylogeny, placement of some taxa remains uncertain as different datasets support competing class-relationships. Traditionally, morphologists have placed Monoplacophora, a group of morphologically simple, limpet-like molluscs as sister group to all other conchiferans (shelled molluscs other than Polyplacophora), a grouping that is supported by the latest large-scale phylogenomic study that includes *Laevipilina*. However, molecular datasets dominated by nuclear ribosomal genes support Monoplacophora + Polyplacophora (Serialia). Here, we evaluate the potential of mitochondrial genome data for resolving placement of Monoplacophora.

**Results:**

Two complete (*Laevipilina antarctica* and *Vema ewingi*) and one partial (*Laevipilina hyalina*) mitochondrial genomes were sequenced, assembled, and compared. All three genomes show a highly similar architecture including an unusually high number of non-coding regions. Comparison of monoplacophoran gene order shows a gene arrangement pattern not previously reported; there is an inversion of one large gene cluster. Our reanalyses of recently published polyplacophoran mitogenomes show, however, that this feature is also present in some chiton species. Maximum Likelihood and Bayesian Inference analyses of 13 mitochondrial protein-coding genes failed to robustly place Monoplacophora and hypothesis testing could not reject any of the evaluated placements of Monoplacophora.

**Conclusions:**

Under both serialian or aculiferan-conchiferan scenarios, the observed gene cluster inversion appears to be a convergent evolution of gene arrangements in molluscs. Our phylogenetic results are inconclusive and sensitive to taxon sampling. Aculifera (Polyplacophora + Aplacophora) and Conchifera were never recovered. However, some analyses recovered Serialia (Monoplacophora + Polyplacophora), Diasoma (Bivalvia + Scaphopoda) or Pleistomollusca (Bivalvia + Gastropoda). Although we could not shed light on deep evolutionary traits of Mollusca we found unique patterns of gene arrangements that are common to monoplacophoran and chitonine polyplacophoran species but not to acanthochitonine Polyplacophora.

**Graphical abstract:**

Complete mitochondrial genome of *Laevipilina antarctica* 
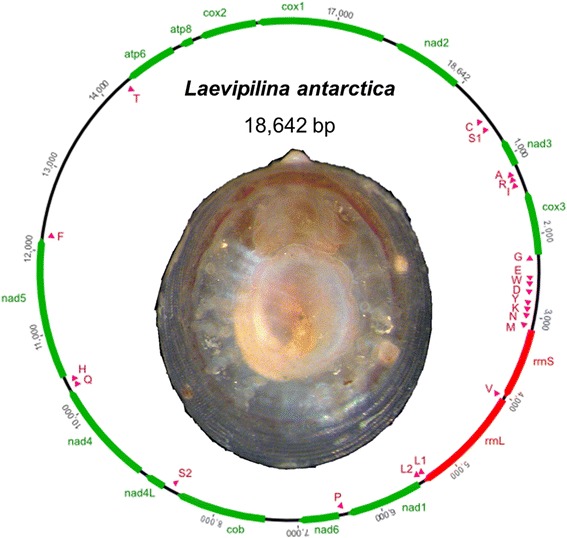

**Electronic supplementary material:**

The online version of this article (doi:10.1186/s12862-016-0829-3) contains supplementary material, which is available to authorized users.

## Background

Mollusca, comprising eight extant classes, has high diversity and an origin that dates back more than 540 million years [1, 2]. One of the most enigmatic classes, Monoplacophora, was thought to be extinct since the Palaeozoic until a living exemplar of *Neopilina galatheae* was found during the Galathea expedition in 1952 [3]. The significance of “living fossil” monoplacophorans for deep molluscan systematics was soon recognized [3], and Monoplacophora (with about 30 recent members called Tryblidia [4]) were central in several palaeontological, morphological and cladistic analyses (e.g., [5–8]) that tried to resolve the phylogeny of Mollusca. These analyses resulted in a number of different phylogenetic placements being hypothesized for Monoplacophora. Under the Conchifera/Aculifera hypothesis, Monoplacophora were traditionally viewed as the sister group to all other conchiferans with and as the sister group of Aplacophora (Caudofoveata + Solenogastres; [9]).

Early molecular analyses based on nuclear ribosomal DNA did not include monoplacophorans [10, 11]. Later analysis of a data set dominated by nuclear ribosomal genes and including all eight extant molluscan classes placed Monoplacophora within Polyplacophora, Serialia [12]. The single 28S sequence from *Laevipilina antarctica* used in that study was a chimera between monoplacophoran and chiton 28S [13], subsequent studies based on the same markers but free of contamination recovered Monoplacophora as sister to Polyplacophora but retained the term Serialia [2, 13, 14]. However, relationships among molluscan classes in these studies were unconventional, recovering Serialia as sister group to bivalves and gastropods, and clustering scaphopods together with aplacophorans and cephalopods. The Serialia hypothesis, which is based on ribosomal DNA dominated data, is provocative, since it challenges traditional taxonomic text-book hypotheses.

Both Aculifera and Conchifera are strongly supported by phylogenomic studies [15–17] and became a new paradigm in molluscan systematics [18–22]; but see [23–25]. Schrödl and Stöger [26] recently emphasized that there is some conflict between the consensus topology (Fig. 1 in [26]), and any of the several phylogenomic [15–17, 27–30] and other nuclear sequence sets [31, 32]. All these molecular datasets cover substantial sequence data, but represent a limited taxon sampling. Smith et al. [16] present the first phylogenomic study including representatives of all eight molluscan classes, and thus it directly addressed placement of Monoplacophora [16, 17]. Although the authors detected many sites in their dataset with weak signal for Serialia and some sites with strong signal for Serialia, the sister group relationship of the one sampled monoplacophoran species and Cephalopoda is clearly favored [16, 17]. A more recent phylogenomic analysis [33] placed the sole monoplacophoran representative employed as the most basal lineage of conchiferans, albeit with low nodal support, but in line with some traditional morphological hypotheses.

**Fig. 1:**
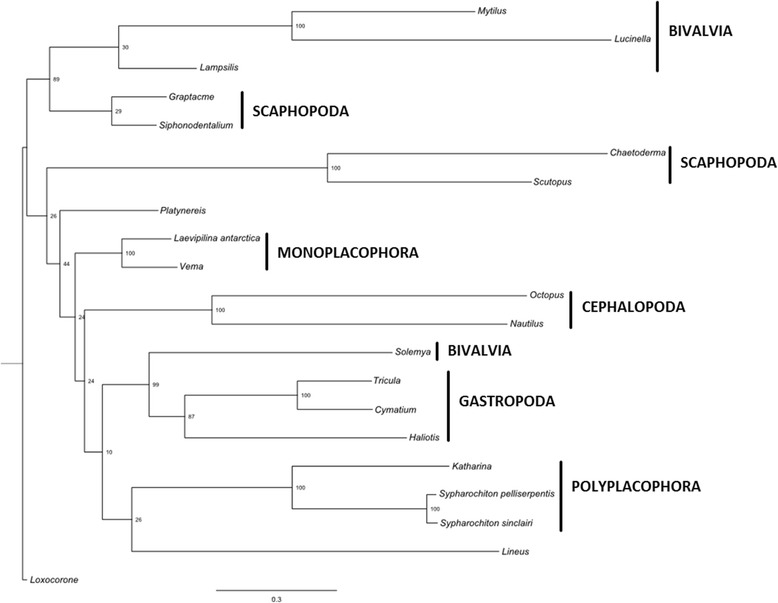
Preferred Maximum Likelihood tree based on the large amino acid dataset and inferred with RAxML-HPC executing 500 bootstrap replicates under the CAT approximation for rate heterogeneity. *Loxocorone* was used to root the tree

An alternative to studying multiple genes is exploring the information content of mitochondrial (mt) genomes [34]. In Metazoa, mitochondrial genomes usually consist of a highly conserved set of 13 protein-coding genes (PCGs), two ribosomal RNAs (rRNAs), and 22 transfer RNAs (tRNAs) [35, 36]. Furthermore, metazoan mtDNA includes at least one (sometimes more in molluscs) non-coding region of which the largest typically contains the control region, the site of initiation for transcription and/or replication [10]. All known mt genomes in molluscs are circular, with orthologs readily identifiable, making them easy to compare. Analyses of mitochondrial protein-coding genes have been successfully used to resolve phylogenetic relationships as for example the affiliation of Sipuncula and Annelida [37–39]. Although the analysis of mitochondrial sequence data provides good resolution in some molluscan subgroups, e.g. Bivalvia [40] or Cephalopoda [41, 42], the resolution for deep molluscan class-relationships is generally poor [35, 43]. Even the analysis of all protein-coding genes of 96 available mt genomes covering six molluscan classes (lacking Monoplacophora and Solenogastres) lacked sufficient phylogenetic signal to robustly resolve relationships among the major lineages of Mollusca [44]. The known problem of increased rates of sequence evolution [45] in some subclades such as bivalves and scaphopods [46] in addition to the Precambrian split of Mollusca from the closest outgroups [2, 30] not surprisingly leads to long-branch attraction ([44]. Taxa showing massive gene rearrangements also show faster nucleotide evolution [26, 34, 44], creating analytical challenges. Stöger and Schrödl [44] recommended analyses of a more representative molluscan taxon set, with fast-evolving taxa at both sequence and gene rearrangement level excluded from analyses. Osca et al. [47] followed this strategy, excluding bivalves and including a second caudofoveate taxon, *Scutopus ventrolineatus*, resulting in an aculiferan/conchiferan topology, although with low support in Maximum Likelihood (ML) analyses. Support for Aculifera is strong for their Bayesian topology, but the clade is nested within outgroup taxa. Plazzi et al. [48] published the first mitogenome of Protobranchia, which are putatively basal lineage of bivalves. This genome appears more conservative relative to the inferred ancestral molluscan and lophotrochozoan arrangements compared to other bivalves, which show greater rearrangement [34, 44]. More recently, mitogenomes of five further chiton species were published in 2014 [49, 50]. According to the authors [49], gene orders are highly congruent with the earlier published mt genome of *Katharina* [35], showing a plesiomorphic arrangement for lophotrochozoans, but this interpretation is not correct.

Here we contribute to the class-level taxon sampling of molluscan mitochondrial genomes by sequencing two Recent monoplacophorans (*Laevipilina antarctica* and *Vema ewingi)* and an almost complete mitogenome of *Laevipilina hyalina*. By generating the first mitogenomes for Monoplacophora our aims were 1) to explore the origin of the enigmatic Monoplacophora, 2) to evaluate whether or not a more balanced taxon excluding rapidly-evolving taxa improved resolution of deep molluscan phylogeny and 3) to compare monoplacophoran gene arrangements with a lophotrochozoan ground pattern [34].

## Results

### General structure/architecture of the monoplacophoran mitogenomes

Mitogenomes of *Vema ewingi* and *L. antarctica* are 17,910 bp and 18,642 bp in length, respectively. Both genomes include the complete set of 37 bilaterian mitochondrial genes: 13 protein-coding genes (PCGs), two ribosomal RNAs (rRNAs), and 22 transfer RNAs (tRNAs). Distribution of PCGs between the two strands is almost equal: ATP synthase subunits (*atp6*, *atp8*) and cytochrome c oxidase subunits (*cox1*, *cox2*, *cox3*), as well as *nad2* and *nad3* are located on the plus strand, whereas NADH dehydrogenase subunits (*nad1, nad4, nad4L, nad5, nad6*) and cytochrome *b* (*cob*) are on the minus strand. Ribosomal genes, *rrnS* and *rrnL*, as well as most of the tRNAs (15 in *L. antarctica*, 16 in *Vema ewingi*) are located on the plus strand. Only seven tRNAs in *L. antarctica* and six in *Vema ewingi* can be found on the opposite (minus) strand. Long-PCR fragments of *L. hyalina* were assembled into 1 contig totaling 15,102 bp and comprising 12 PCGs (*atp8* is missing), both rRNAs and 16 of 22 tRNAs (*trnT, trnC, trnW, trnG, trnH, trnE* are missing). We detected two copies of *trnK* in *L. hyalina*. One copy with a lower e-value (5.223e-05) is located within the tRNA complex *DYKNM* and the second *trnK* with an e-value of 0.6443 is adjacent to *cox2*. In comparison, that *trnK* with a lower e-value is more probable. Both copies of *trnK* show typical cloverleaf secondary structures, similar to that of the two other monoplacophoran *trnK* structures, and the typical anticodon for lysine (UUU), so both copies are potentially functional. All PCGs that could be detected by MITOS are evenly distributed between both strands whereas *rrnS* and *rrnL* are exclusively located on the positive strand. Twelve tRNAs can be found on the plus strand, five are on the minus strand.

For *L. antarctica*, the GC content of the complete mitochondrial genome is 35.5%. GC content of individual PCGs ranges between 33.9% in *atp8* and 39.8% in *cox2* and values for ribosomal RNAs are slightly below the average of the complete genome with 34.4% for *rrnS* and 31.7% for *rrnL*. Transfer RNAs show considerable variation in their GC content with values ranging from 16.1% (*trnH*) to 46.8% (*trnY*). The GC content of the complete mitochondrial genome of *Vema ewingi* is 36.7% with a GC content of PCGs between 33.9% (*nad3*) and 40.4% (*nad6*). Both ribosomal RNAs have a value of 33.8% and tRNAs range between 17.5% (*trnH*) and 55.6% (*trnY*). GC content of the mitogenome of *L. hyalina* is 38.8%. GC content of PCGs is minimum 36.0% in *nad3* and maximum 45.8% in *cox2.* For ribosomal RNAs the GC content is 37.5% for *rrnS* and 34.3% for *rrnL*, within tRNAs range from 22.7% in *trnS2* to 50.0% in *trnY*.

Based on the MITOS results, we identified 28 non-coding regions (NCR) within the mitogenome of *L. antarctica*. Six are less than 10 bp long, 16 are between 10 and 100 bp in length and only six are larger than 100 bp. The largest NCR between *trnF* and *trnT* is 2012 bp long and contains a pattern with the regular expression TATA[TC]ATATATA[GT]A[CT][AT][TA][AT][TCG][GC], we refer to that pattern hereinafter as motif 1. Motif 1 includes an (AT)_6_ repetition (see Table 1). Moreover, some repetitive motifs occur in that NCR (not shown). Motif 1 is additionally detected within the NCR between *trnG* and *trnE* (181 bp) with (AT)_7_. A second motif with the regular expression CCTCGAAATCGTTGCATC (motif 2, Table 1), is visible in the NCR between *nad2* and *trnC* (478 bp)*.* Moreover the NCR between *trnF* and *trnT* includes remains of *atp6*. In the NCR between *nad6* and *cob* MITOS detects residual sequence parts of *nad6*.

**Table 1:** Table shows motifs 1 and 2, their location in the mitogenome and the specific motif sequence

Motif no.	Occurrence	NCR border	Starting position within NCR	Motif sequence
1	*L. antarctica*	trnG/trnE	55	TATATATATATAGATATATG
1	*Vema ewingi*	trnG/trnE	78	TATATATATATATACATATG
1	*L. antarctica*	trnF/trnT	893	TATATATATATAGACTATCG
1	*Vema ewingi*	trnF/trnT	898	TATACATATATATACTTAGC
2	*L. antarctica*	nad2/trnC	23	CCTCGAAATCGTTGCATC
2	*Vema ewingi*	nad2/trnS1	22	CCTCGAAATCGTTGCATC

In *Vema ewingi* we found 27 non-coding regions; five regions are less than 10 bp long, 18 are 10-100 bp long and four are larger than 100 bp. The largest NCR between *trnF* and *trnT* (2287 bp) as well as the NCR located between *trnG* and *trnE* (151 bp) contain motif 1, which is already described for *L. antarctica*. Between *trnF* and *trnT* the motif contains (AT)_6_ with a discontinuity of one (CA), and between *trnG* and *trnE* we count (AT)_9_ (Table 1). Motif 2 was detected in the NCR between *nad2* and *trnS1* (108 bp) (Table 1). Moreover, repetitive motifs are visible in this largest NCR between *trnF* and *trnT* of *Vema ewingi* (not shown).

Within the partial mitogenome of *L. hyalina* we found 21 NCRs, one of which is less than 10 bp long. Sixteen regions are 10 to 100 bp and four are more than 100 bp in length. Within the NCR between *cox1* and *trnK* (299 bp) motif 1 with (AT)_10_ is visible.

The largest NCRs of *L. antarctica* and *Vema ewingi* are located between *trnF* and *trnT* in both mtDNAs and in both NCRs the congruent motif 1 which includes AT-repetitions occurs at almost the same relative positions (Table 1). This motif 1 is recovered in a second NCR in each mitogenome again at congruent relative positions. Motif 2 can be found in NCR between *nad2* and *trnC* in *L. antarctica* and in NCR between *nad2* and *trnS1* of *Vema ewingi.* This motif 2 is located at almost identical relative positions (Table 1). Neither comparisons of these two NCRs to the BLAST nucleotide database results in any similarities to gene regions of other taxa nor are the 2D-foldings informative, which were computed in Geneious with default parameters.

Comparing the relative gene borders of the non-coding regions of the three monoplacophoran species, we discovered 13 NCRs that are embedded between the same genes in all three monoplacophoran genomes (Fig. 3). This number might be even higher since we do not know all NCR borders of *L. hyalina*. Identical positions of NCRs relative to gene order between *L. antarctica* and *Vema ewingi* are 11 whereas only one NCR has the same position between both *Laevipilina* species (Fig. 3). All three monoplacophoran species appear to share two NCRs with the cephalopod *Nautilus* [51]. This is NCR between *cox1* and *nad2*, and NCR between *nad1* and *trnP* (Fig. 3).

We detected six overlapping regions that occur in all three monoplacophoran mt genomes. These overlaps are located between gene pairs *trnY*/*trnK*, *trnM*/*rrnS*, *rrnS*/*trnV*, *rrnL*/*trnL1*, and *trnP*/*nad6* (Fig. 3). Two pairs, *trnV*/*rrnL* and *rrnL*/*trnL1*, are overlapping with more than 25nts according to the MITOS annotation output.

### Gene order within Monoplacophora

Gene arrangements of *L. antarctica* and *Vema ewingi* are shown in Fig. 3. They appear in two clusters (cluster means a group of genes in the following), this is *trnT-atp6-atp8-cox2-cox1-nad2-trnC-trnS1-nad3-trnA-trnR-trnI-cox3-trnG* on one strand and *trnE-trnW-trnD-trnY-trnK-trnN-trnM-rrnS-trnV-rrnL-trnL1-trnL2-nad1-trnP-nad6-cob-trnS2-nad4L-nad4-trnQ-trnH-nad5-trnF* on the opposite strand for *L. antarctica* (Fig. 3). The difference in *Vema ewingi* is the position of *trnC,* which is not located between *nad2* and *trnS1* as in *L. antarctica*, but is found within the tRNA complex *GEWDCYKNM*. The two gene clusters, *nad4/nad4L* and *atp6/atp8* are known to appear adjacent to each other in many animals [40, 52], which is detected here, too.

Within the partial mt genome of *L. hyalina* we observed a very similar gene order and orientation as in *L. antarctica* and *Vema ewingi*, although there are some differences (aside from missing genes). *TrnC* as well as tRNAs *GEW* are missing in the cluster *GEWD[C]YKNM* in the gene order of *L. hyalina* (Fig. 3). Though *trnK* is present within that complex, a second *trnK* with a much more reliable e-value appears adjacent to *cox2. TrnH*, adjacent to *trnQ* in *Vema* and *L. antarctica,* is missing in *L. hyalina*, as well as *trnF* and *atp8*.

The gene order in monoplacophoran PCGs and rRNA genes investigated herein is highly similar, therefore we summarize these arrangements and refer to it as the monoplacophoran plesiomorphic state.

### Gene order within Polyplacophora

In addition to the mitogenome of the black chiton *Katharina* [35] five more chiton mitogenomes are available now [49, 50]. The three acanthochitonine mt gene arrangements (*Cryptochiton*, *Cyanoplax*, *Nuttalina*) are in line with the *Katharina* arrangement except the two tRNA complexes *KARNI* and *MCYWQGE*, which are present in *Katharina* and *Cryptochiton*. Both complexes appear in inversed orders in *Nuttalina* and *Cyanoplax*. Although mitogenomes of the chitonine taxa *Sypharochiton pelliserpentis* and *S. sinclairi* have already been published their gene order is not thoroughly examined [49]. The authors claim that the gene arrangements of their chitonine species resemble that of other chitons, but did not show the actual arrangement, so we have reexamined these mitogenomes (Fig. 3). Both *Sypharochiton* mitogenomes are congruent to each other in their gene arrangement but *contra* [49] the gene order is not “almost identical to that found in *Katharina tunicata*” ([49], Fig. 3 herein). The genes of *Sypharochiton* are arranged in the two clusters of genes that are already described for Monoplacophora (Fig. 3). Moreover, these two clusters have identical orientation as in the monoplacophoran arrangement (Table 4). Differences to the monoplacophoran gene order are restricted to the two tRNA complexes: one is *INRAK* in *Sypharochiton*, the second is *EGQWYCM*, which are exactly inverse to the *Katharina* order (Fig. 3, Table 4), but congruent to the order of *Nuttalina* and *Cyanoplax*.

### Phylogenetic analyses

Our initial taxon set based on the amino acid alignment of all protein-coding genes includes 18 molluscs and three lophotrochozoan outgroup taxa (Table 5, Fig. 1, Additional file 1: Figure S1). The entoproct *Loxocorone* was used to root the tree as it represents the most distant related of the non-mollusc taxa employed [33]. Maximum Likelihood (ML) analysis of this taxon set recovers Mollusca as non-monophyletic with *Platynereis* (Annelida) and *Lineus* (Nemertinea) nested within Mollusca. Monoplacophora, Polyplacophora, Caudofoveata, and Cephalopoda were recovered monophyletic with maximal bootstrap support (bs) whereas support for gastropod monophyly was moderate (bs = 87%) and support for scaphopod monophyly was weak (bs = 29%). Relationships among higher level taxa were generally poorly supported. Also, Scaphopoda together with three non-protobranch bivalves form a moderately well-supported clade (bs = 89%; Fig. 1, Table 2).

**Table 2:** Table gives an overview on all Maximum Likelihood (pre-) analyses and the resulting molluscan hypotheses; taxon sets aa-1 – aa-11 are based on amino acis data, taxon sets nuc-1 – nuc-11 are based on nucleotide datasets; main analyses based on aa-1 and aa-2 were additionally analyzed with Phylobayes which is indicated in the first column; Numbers are bootstrap support values of the corresponding hypothesis that appeared in that analysis, numbers followed by “pp” are posterior probabilities of the Phylobayes analysis; “-“means that the hypothesis did not appear in that analysis

Taxon set	Inclusion/exclusion of taxa	Monophyletic Mollusca	Aculifera	Conchifera	“Diasoma” (non-protobranch Bivalvia + Scaphopoda)	Monoplacophora + Cephalopoda	“Pleistomollusca” (Solemya + Gastropoda)	Serialia
aa-1	initial and largest taxon set, comprising 18 molluscan taxa and 3 lophotrochozoan outgroup taxa	-	-	-	81	-	100	-
aa-1 Phylobayes	initial and largest taxon set, comprising 18 molluscan taxa and 3 lophotrochozoan outgroup taxa, analyzed with Phylobayes	-	-	-	-	-	Bivalvia + Gastropoda: 0.99 pp	-
aa-2	initial taxon set except non-protobranch Bivalvia and outgroups reduced to *Platynereis* (Annelida) only	0	-	-	-	-	100	-
aa-2 Phylobayes	initial taxon set except non-protobranch Bivalvia and outgroups reduced to *Platynereis* (Annelida)only, analyzed with Phylobayes	-	-	-	-	-	0.96 pp	-
aa-3	initial taxon set except non-protobranch Bivalvia, Scaphopoda and outgroups reduced to*Platynereis* (Annelida) only	0	-	-	-	-	100	-
aa-4	aa-1 without any outgroups	-	-	-	8	-	-	-
aa-5	aa-2 without any outgroups	-	-	-	-	-	-	-
aa-6	aa-3 without any outgroups	-	-	-	-	-	-	-
aa-7	aa-1 plus *Scutopus*	-	-	-	-	11	99	-
aa-8	aa-2 plus *Scutopus*	0	-	-	-	-	-	-
aa-9	aa-1 plus *Scutopus* and two *Sypharochiton* species	-	-	-	-	-	99	-
aa-10	aa-2 plus *Scutopus* and two *Sypharochiton* species	0	-	-	-	-	99	-
aa-11	aa-10 *Solemya* excluded	0	-	-	-	-	-	-
nuc-1	initial and largest taxon set, comprising 18 molluscan taxa and 3 lophotrochozoan outgroup taxa	-	-	-	-	-	-	-
nuc-2	initial taxon set except non-protobranch Bivalvia and outgroups reduced to *Platynereis* (Annelida) only	0	-	-	-	-	-	100
nuc-3	initial taxon set except non-protobranch Bivalvia, Scaphopoda and outgroups reduced to *Platynereis*(Annelida) only	0	-	-	-	-	-	56
nuc-4	nuc-1 without any outgroups	-	-	-	92	-	-	100
nuc-5	nuc-2 without any outgroups	-	-	-	-	-	-	-
nuc-6	nuc-3 without any outgroups	-	-	-	-	-	-	100
nuc-7	nuc-1 plus *Scutopus*	-	-	-	-	-	-	100
nuc-8	nuc-2 plus *Scutopus*	0	-	-	-	-	-	100
nuc-9	nuc-1 plus *Scutopus* and two *Sypharochiton* species	-	-	-	-	-	-	-
nuc-10	nuc-2 plus *Scutopus* and two *Sypharochiton* species	0	-	-	-	-	-	-
nuc-11	nuc-10 *Solemya* excluded	0	-	-	-	-	-	-

Phylobayes analysis of this dataset recovered a topology that is unresolved at its base. All classes of Mollusca except Scaphopoda were recovered monophyletic with strong support (posterior probabilities, pp = 0.99-1.00). Pleistomollusca was also strongly supported (pp = 0.99) and Monoplacophora was recovered sister to Caudofoveata (albeit with weak support by Bayesian standards, pp = 0.84; Additional file 1: Figure S1, Table 2).

**Fig. 2:**
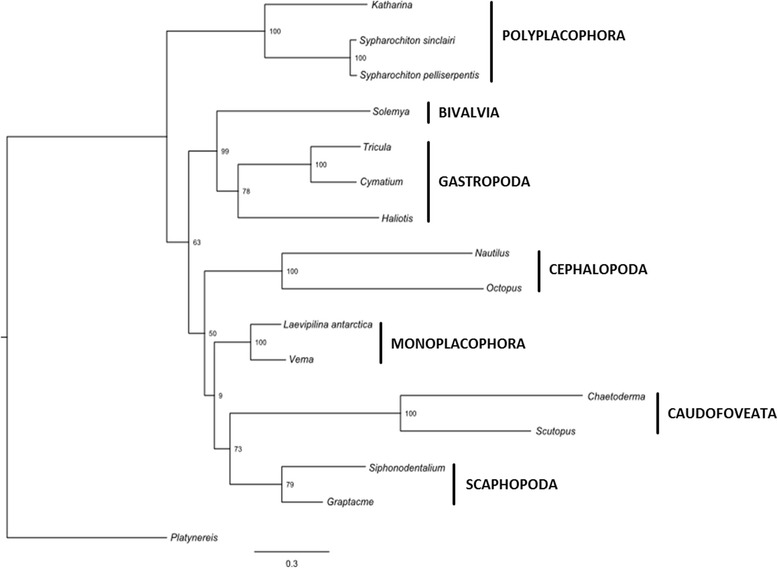
Maximum Likelihood tree based on the amino acid dataset without the two outgroup taxa *Lineus* and *Loxocorone* and the reduction of bivalve taxa to the protobranch taxon *Solemya.* Tree was inferred with RAxML-HPC executing 500 bootstrap replicates under the CAT approximation for rate heterogeneity. *Platynereis* was used to root the tree

Exclusion of the outgroup taxa *Lineus* and the more distant outgroup *Loxocorone* and the reduction of bivalve taxa to the protobranch taxon *Solemya*, which is the most basal bivalve group, lead to a ML topology with a strongly supported Pleistomollusca (bs = 99) and a moderately supported sister group relationship of Caudofoveata and Scaphopoda (bs = 73; Fig. 2). Phylobayes analysis of this trimmed down dataset (Additional file 1: Figure S1, Table 2) yielded similar results with Scaphopoda (pp = 0.72) being the most weakly supported class and Pleistomollusca recovered (pp = 0.96).

In our test on saturation of the alignments TreSpEx calculated *cox1* as the least saturated and *nad6* as the most saturated. There is a gradual decline in the slope value from the best to the worst so cutting out particular genes does probably not improve the tree topology. BaCoCa measures rate heterogeneity and again detects *cox1* as the “best” gene but there is a gradual decline. *Platynereis* and *Nautilus* are the most compositionally heterogeneous taxa in the datasets but not extremely so. Overall, we were not able to identify certain genes or taxa that are particularly problematic.

Hypothesis testing using the Shimodaira-Hasegawa (SH) test and the Approximately Unbiased (AU) test failed to reject Aculifera, Conchifera, Monoplacophora as the sister taxon to the rest of Conchifera, Monoplacophora sister to Cephalopoda, Serialia, or Testaria as being significantly less likely than the most likely tree recovered in either of the two ML analyses (Table 3). Hypothesis testing was performed on both main datasets (aa-1 and aa-2 in Table 2).

**Table 3:** Results of SH and AU hypothesis testing

Analysis	Constraint	Log-likelihood	AU-test p-value	SH-test p-value
aa-1	Unconstrained	−99817.64	0.852	0.935
aa-1	Aculifera monophyletic	−99837.74	0.113	0.392
aa-1	Conchifera monophyletic	−99854.34	0.113	0.113
aa-1	Monoplacophora sister to rest of Conchifera	−99859.16	0.069	0.089
aa-1	Monoplacophora sister to Cephalopoda	−99825.77	0.467	0.704
aa-1	Serialia monophyletic	−99839.30	0.062	0.354
aa-1	Testaria monophyletic	−99860.90	0.053	0.083
aa-2	Unconstrained	−99817.64	0.854	0.940
aa-2	Aculifera monophyletic	−99837.74	0.130	0.390
aa-2	Conchifera monophyletic	−99854.34	0.118	0.120
aa-2	Monoplacophora sister to rest of Conchifera	−99859.16	0.066	0.092
aa-2	Monoplacophora sister to Cephalopoda	−99825.77	0.449	0.703
aa-2	Serialia monophyletic	−99839.30	0.063	0.353
aa-2	Testaria monophyletic	−99860.90	0.053	0.083

## Discussion

### Gene order

**Fig. 3:**
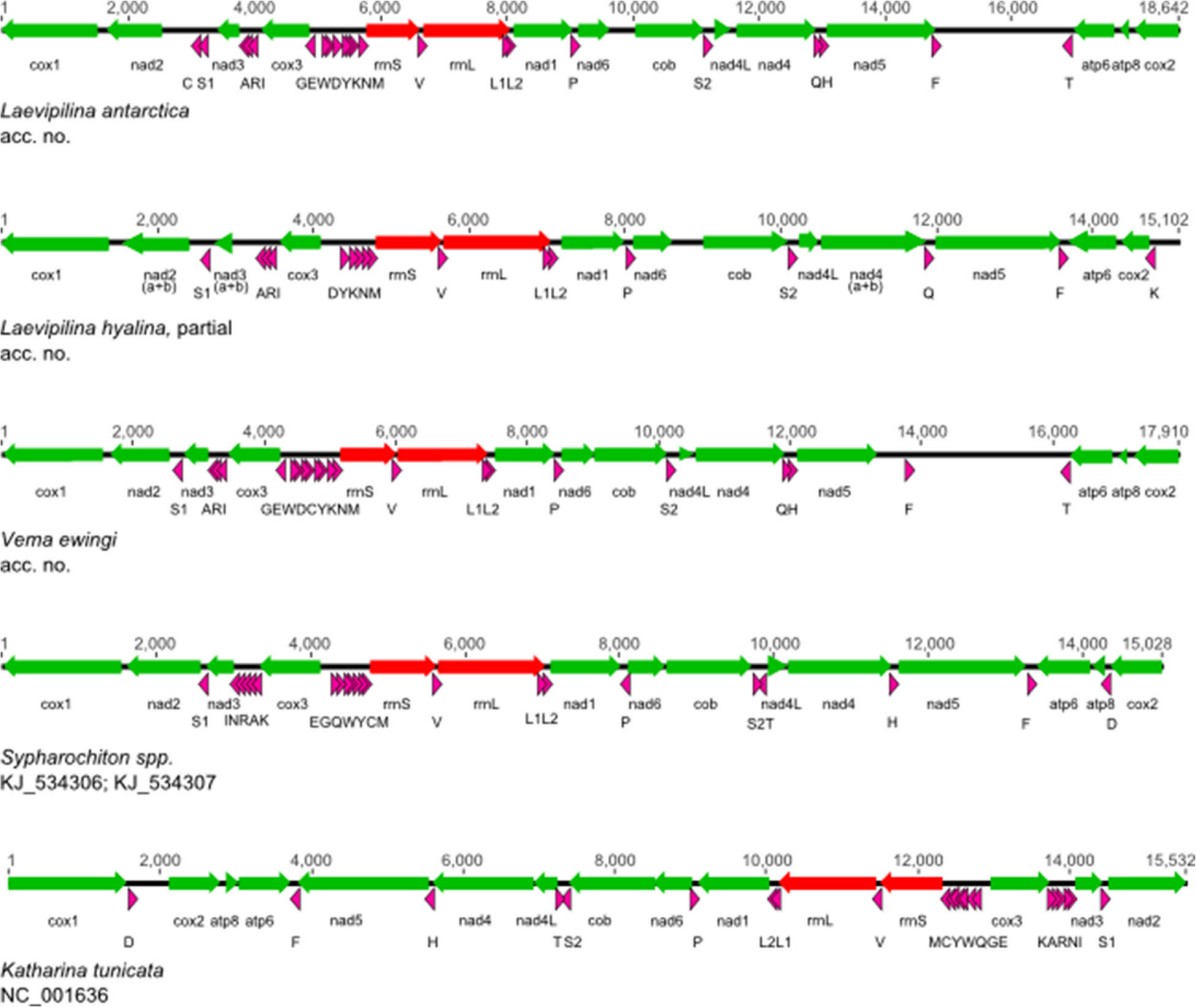
Gene arrangements of selected molluscan taxa; arrangements are annotated with MITOS and linearized and rotated to *cox1* for display reasons. Gene lengths of coding and non-coding regions correspond to relative lengths of the genomes. The directions of the genes are given by arrows. Green arrows indicate protein coding genes (PCGs); red arrows indicate ribosomal genes (rRNAs); pink arrows indicate transfer RNAs (tRNAs), which are named corresponding to the one-letter code. *Sypharochiton sinclairi* and *S. pelliserpenti*s showed identical gene order

The gene arrangement of Monoplacophora revealed herein is either highly conserved or the taxa here recently diverged from each other. *L. antarctica* and *Vema ewingi* differ only in the position of *trnC* which is adjacent to *trnS1* in *L. antarctica* but is embedded in the tRNA complex *GEWDCYKNM* in *Vema ewingi* (Fig. 3).

Presence of two conserved gene blocks was confirmed in these monoplacophoran species (Table 4). One conserved block, *rrnS-rrnL-nad1-nad6-cob,* was defined previously for Lophotrochozoa [34], and the second block that is putatively conserved in Lophotrochozoa, *nad4L-nad4-trnH-nad5*, could be detected adjacent to *rrnS-rrnL-nad1-nad6-cob* (shown as combined cluster 2 in Table 4), although in a somehow aberrant appearance concerning tRNAs, since in *L. antarctica* and *Vema ewingi* there is *trnQ* nested between *nad4* and *trnH* and in *L. hyalina trnH* is missing (Fig. 3). The part of yet another lophotrochozoan conserved gene block (*cox3-nad3-nad2-cox1-cox2-atp8-atp6*) usually appears in the forward direction. In our monoplacophoran species the part *nad2-cox1-cox2-atp8-atp6* is inverted (Table 4). In *L. hyalina atp8* is missing but *trnK* is included. Presence of these conserved lophotrochozoan gene blocks and a relatively high percentage of divergence between the PCGs of the monoplacophoran species (22.4%) leads to the assumption that gene order in Monoplacophora is conserved.

**Table 4:** Directions of PCGs and rRNAs in the two clusters; tRNAs are not considered. Based on the lophotrochozoan ground pattern [34] we find two evolutionary lines. One is evident in *Katharina,* as well as in *Octopus,* with an inversion of PCGs in cluster 2. From this derived arrangement we can infer the *Nautilus* gene order with a “simple” translocation of rRNAs. The second line is an inversion of cluster 1 of the lophotrochozoan ground pattern, which leads to the monoplacophoran (and the *Sypharochiton*) pattern of gene arrangement. We could not detect this arrangement of PCGs in another lophotrochozoan group so far (see e.g., [80])

	Cluster 1: cox3-nad3-nad2-cox1-cox2-atp8-atp6	Cluster 2: rrnS-rrnL-nad1-nad6-cob-nad4L-nad4-nad5	Remarks
Lophotrochozoan ground pattern (Bernt et al. [34])	→	→	
Monoplacophoran plesiomorphic state	←	→	Cluster 1 missing *atp8* in *L. hyalina* as it was not sampled
*L. antarctica*	←	→	
*Vema ewingi*	←	→	
*L. hyalina*	←	→	Cluster 1 misses *atp8*
*Sypharochiton spp.*	←	→	
*K. tunicata*	→	←	

A potential synapomorphy for Mollusca [44], aggregation of *trnG-trnE* with the tRNA complex *MCYWQ,* is present in Monoplacophora, although the complex is reversed in its order (Fig. 3). A second tRNA complex that appears frequently in Lophotrochozoa is *KARNI* [44]. Within our monoplacophoran taxa we instead find *ARI* which is also present in the caudofoveate *Chaetoderma*. A clade of caudofoveates and monoplacophorans is recovered by some of our sequence analyses, but not by any other analyses including nuclear data (for review see [26]); we thus assume that congruency in the tRNA order *ARI* is convergent.

Focusing on the gene order of protein-coding genes (PCGs) and ribosomal RNAs, the ancestral state for both PCG clusters is forward in the lophotrochozoan ground pattern (cluster 1 and 2, see Table 4). Within Mollusca, the order of PCGs that is observed in *Katharina* and other Acanthochitonina [35, 50] is hypothesized to represent the ancestral arrangement for at least molluscs, since this arrangement is recurring with no or almost no modifications in other molluscan classes [44]. In reference to the lophotrochozoan pattern, we show that the orientation of cluster 1 of the Acanthochitonina gene order is ancestral, whereas cluster 2 is derived (Table 4). This order is opposite in Monoplacophora: Their gene orders reflect a derived orientation for cluster 1, but the plesiomorphic state for cluster 2, which appears to be a unique condition among lophotrochozoans. We confirm a plesiomorphic gene arrangement in Acanthochitonina but a monoplacophoran-like derived gene order in Chitonina (Table 4). Rearrangements of PCG clusters are considered to be rare events, and thus are given high phylogenetic significance [51, 52]. Accordingly, the uniquely derived arrangement of cluster 2 could be interpreted as a synapomorphy, supporting Serialia; because of the undisputed monophyly of Polyplacophora, the heterogeneous arrangement within chitons implies homoplasy. Unfortunately, no information is available on mitogenomes of the Lepidopleurida, the morphologically most plesiomorphic chiton clade [53, 54]. Under the Aculifera-Conchifera concept we find this derived condition of gene order in some but not all members of both major clades, also implying convergence within Mollusca. Such convergent rearrangements of large PCG complexes have rarely been detected in invertebrates [52] but not in vertebrates [55]. One such example is known from Caenogastropoda, which shares a congruent gene order of PCGs with the nemertean *Lineus* [44]. We could not find any similar examples within molluscs in the literature and we anticipate that denser sampling may reveal more cases.

### Gene architecture

Mitogenome lengths of *L. antarctica* and *Vema ewingi* are consistent with other molluscan mitochondrial genomes, which range between 13.6 kb in *Biomphalaria* (Gastropoda) to 31.5 kb in *Placopecten* (Bivalvia) [44]. Nevertheless, both range at the upper bound of animal mtDNA length, which is typically less than 20 kb [56]. Both mitogenomes contain the complete gene complement of a typical bilaterian mitogenome [52]. *L. hyalina* lacks *atp8* and six tRNAs. *Atp8* is conserved in just a short fragment at the 5′ region [36, 57], which makes it rather difficult to identify. That might explain the absence of *atp8* in *L. hyalina* since that gene is not located at the boundaries of the contig sequence that was used as input for MITOS, where we would expect missing data in an incomplete mitochondrial genome.

We detected two copies of *trnK* in *L. hyalina,* both highly similar to the *trnK* of *L. antarctica* and *Vema ewingi* in their structure. Duplication of tRNAs is not uncommon and has been reported before (e.g., [37]). A partial inversion of at least *cox1-cox2-trnK* of a conserved lophotrochozoan gene complex could explain the duplicated *trnK* detected in *L. hyalina*, since MITOS additionally detected relics of *cox1* in a row with *trnK* and *cox2* in that individual arrangement. This could also indicate a tandem duplication random loss event.

The three monoplacophoran mitogenomes analyzed herein exhibit almost the same number of non-coding regions; 21 in the incomplete mtDNA of *L. hyalina* and 27 and 28 in *Vema ewingi* and *L. antarctica,* respectively. Several non-coding regions are larger than 100 bp, distributed throughout the genomes and differing substantially in lengths within the same genome. This occurs frequently in molluscan mitogenomes. For example, in the class Gastropoda, some families possess many small NCRs [58, 59], as well as in Cephalopoda, which show intergenic regions that may be longer than 900 bp [51]. *Katharina* (Polyplacophora) also has several NCRs [35], and the bivalve taxon *Placopecten* contains NCRs up to 10,000 bp [57]. Almost half of the NCRs in Monoplacophora are located between the same genes in all three mtDNAs. *L. antarctica* shares more relative gene boundaries of NCRs with *Vema ewingi* than with *L. hyalina;* this is unexpected since it suggests a closer relationship of *L. antarctica* to *Vema ewingi* than to *L. hyalina*, but this might also be due to information missing in *L. hyalina*. The congruent relative location of two NCRs found in *Nautilus* and Monoplacophora with two identical or even highly similar sequence motifs might be synapomorphic and thus indicate common ancestry for monoplacophorans and cephalopods as it is proposed by Smith and colleagues [16, 17]; however, the motifs are very short and could also be either plesiomorphic or convergent.

Each of the two complete mitogenomes of *L. antarctica* and *Vema ewingi* has its largest NCR between *trnF* and *trnT* (see Fig. 3). These NCRs are 2012 bp and 2287 bp long respectively and both contain the AT-rich motif 1 that is almost identical in both mitogenomes concerning nucleotide composition, length, and position within the NCR (Table 1). A very similar motif is visible in *L. hyalina* in the NCR between *trnK* and *cox1* (Table 1). The long and unassigned regions could be the potential origins of transcription of our monoplacophoran mtDNAs since AT-rich motifs are usually evidence for the control region of mitogenomes [59, 60]. Several other repetitive motifs are visible in the largest NCRs of *L. antarctica* and *Vema ewingi,* which provide even more evidence that this region is the control region. Motif 1 is repeated between *trnG* and *trnE* in *L. antarctica* and *Vema ewingi*, again with almost congruent starting points and very similar positions within the NCRs (Table 1, Fig. 3). We hypothesize that the initiation region was partially duplicated to have two starting points for the replication process which would lead to an increased transcription rate as was suggested for cephalopods before [61]. Although we found evidence for the potential control region in *L. hyalina*, too, we were not able to detect its duplication in this incomplete mt genome.

MITOS annotated fragmentary *cox1* in *L. hyalina* and parts of *atp6* in *L. antarctica* in the potential initiation regions. These protein-coding gene fragments are located near their functional copies. A possible scenario could be that part of the mitogenome, consisting minimally of the respective PCGs, was duplicated, and this is still visible in both *Laevipilina* individuals through residual PCG fragments. These duplicated copies might be in the process of being lost. Whether in *Vema ewingi* the loss is already finished, or the duplication event never took place is not clear. Nevertheless, we identified a region of accelerated rearrangement rate and this is third indication for locating the origin of replication in these NCRs in *Laevipilina*. Such a control region is usually described as the longest non-coding region within the mitogenome that is rich in AT, often including repetitive motifs, and seems to be a hotspot for rearrangements [59, 62]. The existence of duplicated control regions or parts thereof could be seen as a similarity for Monoplacophora and Cephalopoda (see [63]), since this feature is not known from other molluscs so far but is observed in other metazoan mitogenomes [64–66].

The second repetitive sequence motif (motif 2), is found in *L. antarctica* in the unassigned region between *nad2* and *trnC* as well as in *Vema ewingi* in the non-coding part between *nad2* and *trnS1* (Table 1)*.* This motif starts in both NCRs at almost the same position. Unassigned regions are known to be extremely variable because they do not underlie any selective pressure. Independent evolution of two identical 18 bp long nucleotide motifs in the same position is unlikely, so this motif is probably an apomorphy inherited from the common ancestor of these two taxa.

### Phylogeny

Several phylogenetic approaches resulted in ambiguous topologies, which were sensitive to taxon sampling. Neither nucleotide nor amino acid taxon sets supported Aculifera (Polyplacohora + Aplacophora) or Conchifera (comprising all other shell-bearing classes), in contrast to Osca and colleagues [47] (see Table 2). A trend in amino acid analyses is the repeated recovery of a highly supported Pleistomollusca, whereas nucleotide based analyses supported Serialia (Table 2). In the data set with 3 non-molluscan outgroups, neither the amino acid nor nucleotide datasets supported the monophyly of Mollusca, which is, however, well-established [2, 15, 16, 27, 28, 32]. Molluscan non-monophyly is a common result of phylogenetic analyses based on mt protein coding genes [34, 44, 46] which was unaffected by the addition of more taxa here (Table 2).

Analyses recovered a monophyletic Monoplacophora and tended to support monophyly of other molluscan classes, except for bivalves. Non-protobranch bivalves have longer branches and rearranged gene orders compared to the protobranch *Solemya*. Such high levels of gene rearrangements were suggested to be linked with high rates of nucleotide substitution [26, 34, 44].

In amino acid datasets, the lamellibranch bivalves cluster as the sister group to scaphopods, but *Solemya* clusters with gastropods (Fig. 1, Table 2). The latter relationship was also recovered by Plazzi et al. 2013 [48] but was interpreted as an artifact due to limited phylogenetic signal in the bivalve lineage of Opponobranchia (including Nuculida and Solemyida). *Solemya* is the only bivalve in our dataset that has its genes arranged on both strands, a fact that leads to different substitution skew between plus and minus strands of the mt genome. Such differences in nucleotide composition might influence phylogenetic analyses and could be an explanation for our diphyletic clustering of bivalve taxa [67, 68].

Pruning non-protobranch bivalves recovers *Solemya* as the sister group to gastropods, i.e. a taxon Pleistomollusca ([15], Fig. 2; Additional files 1 and 2: Figures S1 and S2) in most amino acid analyses. Excluding the remaining protobranch bivalve, *Solemya*, from our analyses did not result in an aculiferan topology (Table 2). That is in contrast to Osca et al. [47] who excluded Bivalvia and recovered Aculifera (although Solenogastres was not sampled) either with poor support (ML) or with strong support but not as part of a monophyletic Mollusca (BI). In the taxon set in Osca et al. [47], Conchifera were lacking Bivalvia, which were pruned, and Monoplacophora.

As Osca et al. [47] recovered Aculifera and Conchifera we expected that adding further, taxa such as protobranchs and monoplacophorans might be beneficial to resolve further aspects of deep molluscan evolution. Within this study we employed different taxon sets to explore the robustness of the data. However, the diversity of topologies recovered herein is striking and suggests there is limited phylogenetic signal in this data. By modifying datasets we recovered several formerly proposed and currently disregarded hypotheses of higher taxa, but never the preferred Aculifera or Conchifera [47].

## Conclusion

This mitogenomic study includes three members of two monoplacophoran genera. Our phylogenetic results of analyzing the protein coding supermatrix of 13 genes of 18 selected molluscan taxa across 7 of 8 classes stay ambiguous. Common and highly accepted molluscan hypotheses as the Aculifera or Conchifera concepts never appear in any of our phylogenetic permutations.

Our finding of unique protein gene arrangements in Monoplacophora and chitonine but not acanthochitonine Polyplacophora is remarkable because it may support the Serialia hypothesis, which is in conflict with the Aculifera/Conchifera hypothesis, but more likely it represents a plesiomorphic genome structure for molluscs. Any topology would imply convergent evolution of identical PCG clusters within Mollusca. On one hand, this clearly weakens the significance of supposedly rare gene rearrangement events and single genome level characters. On the other hand, this demonstrates the existence of further genome level characters that may become useful if mitogenomes are explored densely over molluscan (and other) taxa. Unfortunately, phylogenetic analyses of the mtDNA provided little information for resolving mollusc phylogeny. Furthermore, we need to expand our yet limited knowledge on mitochondrial evolution and data from the molluscan class Solenogastres (=Neomeniomorpha) is still lacking. High throughput sequencing as used here is a powerful and accurate way to add further mitogenomes of taxa that are small or with limited material available.

## Methods

### Preparation of *Vema ewingi*

*Vema ewingi* was collected on R/V “Dimitry Mendellev” at 8°S 81°W in 5800 m depth. DNA was extracted and purified using the Qiagen DNeasy kit (Qiagen, Hilden, Germany) following the manufacturer’s protocol. DNA concentration was measured using a Qubit with the double-stranded DNA broad range kit. DNA quality was evaluated using a 1% SB agarose gel. Gel electrophoresis revealed that the DNA was degraded with an average fragment size of around 500 bp. However, some large fragments of DNA up to around 10,000 bp were present.

An Illumina Nextera (Illumina, San Diego, CA, USA) library was prepared following the manufacturer’s protocol. However, the resulting library had a low size distribution because the template DNA was degraded. Additional attempts were made to prepare Nextera libraries using more template DNA than recommended by the Illumina protocol. This produced better quality libraries based on size distribution with the optimal library using four times the recommend amount or 200 ng total.

Sequencing was conducted using a 2 × 250 bp paired-end (PE) v2 kit on the Illumina MiSeq at Auburn University. The *Vema* libraries were sequenced in parallel with libraries for other projects with around eight dual-indexed libraries sequenced at a time. Several attempts at sequencing various *Vema* Nextera libraries were made using different amounts of template DNA, combining all of the *Vema* genomic data collected to that point, and assembling the paired-end reads using Ray 2.2.0 with a k-mer of 31 on the Auburn University SkyNet server never yielded a complete mitochondrial genome.

Therefore, we abandoned the Nextera approach and prepared libraries using the NEB Next Ultra kit (New England Biolabs, Ipswich, MA, USA) for Illumina sequencing. As the DNA was already degraded to an average size of around 500 bp, no shearing was necessary. End-repair, adapter ligation, and barcode incorporation via PCR were conducted following the manufacturer’s protocol. As above, sequencing was conducted using a 2 × 250 bp paired-end (PE) v2 kit on the Illumina MiSeq at Auburn University. Again, around eight indexed libraries were sequenced at a time and after two runs, a complete mitochondrial genome could be assembled for *Vema*.

In order to identify the complete mitochondrial genome, the assembly was searched against a nucleotide BLAST database constructed from the complete mitochondrial genome of *Katharina tunicata* (Polyplacophora) using BLASTN and TBLASTX using an e-value cutoff of 0.01.

### Preparation of *Laevipilina antarctica*

Total genomic DNA was extracted from a piece of tissue of one specimen of *Laevipilina antarctica* (ZSM-Mol-20090330, DNABANK-Mol-MS-016), which was collected during the expedition with R/V Polarstern in Antarctica, using the NucleoSpin Tissue Kit (Macherey-Nagel, Düren, Germany) following the instructions in [69].

Ten nanogram of DNA was used for multiple strand replacement based DNA amplification using the illustra GenomiPhi V2 DNA Amplification Kit (GE Healthcare Life Sciences, Freiburg, Germany) using the manufacturers instruction followed by standard ethanol precipitation. Subsequently the DNA was purified using the Qiagen MinElute system (Qiagen, Hilden, Germany), DNA concentration was determined using the Qubit® 2.0 Fluorometer. 1 μg of DNA was used to create a standard fragment DNA sequencing library with the TruSeq DNA Sample Preparation Kit v2 (Illumina, San Diego, CA, USA); the experimental average insert size was 250 bp. Two lanes of 101 bp paired-end-reads were sequenced on the Ilumina HiSeq2000 system. About 90 Gigabasepairs (Gbp) were obtained. These were filtered for quality, PCR duplicates, and adaptor sequences and corrected using SOAPfilter_v2.0 (https://github.com/tanghaibao/jcvi-bin/blob/master/SOAP/SOAPfilter_v2.0) using default settings. We subsetted 5–200 million paired reads in K-mer iterations of 23–99 and using various parameters for mitogenome assembly using SOAPdenovo2 [70]. The best assembly of the complete mitogenome was discovered using 50 million paired reads and settings other than default –R –u.

### Preparation of *Laevipilina hyalina*

Total genomic DNA was extracted from a single specimen collected off California [13] using the Qiagen DNeasy kit (Qiagen, Hilden, Germany), following manufacturer’s protocols. Standard PCR protocols were used to generate sequences from Cytochrome c oxidase I (COI), 16S rDNA (16S) (see [13]) plus Cytochrome oxidase B (cob) using universals 424f + 876R [71] and Cytochrome c oxidase III (COIII) [72]. All amplifications were done using illustra PuReTaq Ready-To-Go PCR Beads (GE Healthcare Life Sciences, Freiburg, Germany) following the manufacturer’s protocols. PCR products were cleaned using USB ExoSAP-IT, and sequenced by Retrogen Inc. (San Diego, CA, USA). Sequencher v4 was used to inspect and trim sequences. Sequences from these mitochondrial genes were used to design *Laevipilina*-specific primers for long PCR amplification. The Primer3 algorithm was used to design these primers [73].

Various primer combinations were tested, and a final set of MCOIf + MCytbr (5′-ATTGGCTGGGGCAGTTACTA-3′ + 5′-TGTGGAGAGGGGTAACAAGG-3′) and MCOX3f + MCOIR (5′-GATGTTTCGGTTGGGATACG-3′ + 5′-AAAGGAACCCGCTCAAGAGT-3′) resulted in two overlapping fragments (approximately 7 kb and 3 kb respectively). All long PCR products were amplified using Platinum Taq DNA Polymerase High Fidelity (Invitrogen, Waltham, MA, USA) following the manufacturer’s specifications. The PCR products were visualized on 1% agarose gels run at 80 V for 90 min. PCR products were cleaned using GelElute Extraction kit (5 Prime, South San Francisco, CA, USA) and outsourced to Engencore (Selah Genomics, Greenville, SC, USA) for sequencing and assembly with the Roche 454 platform and Newbler v2.3.

### Annotation of mitogenomic consensus sequences

Mitogenomic sequences were filtered from the whole genome assemblies via BLAST searches and by alignment to known sequences of mitochondrial genes. The MITOS web server [74] was used to annotate mitogenomic data of *L. antarctica* and *Vema ewingi* as well as the partial consensus sequence of *L. hyalina.* Mitogenomic consensus sequences of the bivalve *Solemya velum* (NC_017612 [48]), the caudofoveate *Scutopus ventrolineatus* (KC_757645 [47]) as well as *Sypharochiton pelliserpentis* (KJ_534307 [49]) and *S. sinclairi* (KJ_534306 [49]) were downloaded from GenBank and newly annotated via the MITOS web server as well. Recommended default parameters [74] and the invertebrate mitochondrial genetic code (translation Table 5) were used for all annotations of protein coding genes, transfer and ribosomal RNAs. Annotated single sequences were imported in Geneious version 6.1.7 to work on GC content, extract and examine non-coding regions as well as overlaps, to visualize secondary structures of tRNAs of special interest (default parameters in Geneious were used), and to compile the different datasets for phylogenetic analyses (please see section “Phylogenetic analyses” for details). As Tomita et al. [75] proposed for non-coding regions in *Loligo*, we conducted BLAST searches of all non-coding regions larger than 100 bp of our three monoplacophoran genomes to find possible similarities to other mt genomes but we did not find any noticeable hits. Moreover, we checked the largest NCRs (>2 kb) of *L. antarctica* and *Vema ewingi* for group II transposons. This phenomenon was found in the annelid *Nephtys* [76] but also in insects [77] and might give an explanation for the unusually long NCRs in our species. We conducted DNA foldings of the non-coding sequences via the Mfold web server under default options, but could not find any similarities to the described secondary structure of *Nephtys* which is described as a central core with six radiating helical domains [76]. Both NCRs were compared to the Dfam database [78], but no hits were detected.

**Table 5:** Table shows all taxa that were used in this study with their corresponding GenBank accession numbers

	Class	Taxon	GenBank acc. no.
Outgroup taxa	Annelida	*Platynereis dumerilii*	NC 000931
	Entprocta	*Loxocorone allax*	NC 010431
	Nemertea	*Lineus viridis*	NC 012889
Mollusca	Bivalvia	*Lampsilis ornata*	NC 005335
		*Lucinella divaricata*	NC 013275
		*Mytilus edulis*	NC 006161
		*Solemya velum*	NC 017612
	Caudofoveata	*Chaetoderma nitidulum*	NC 013846
		*Scutopus ventrolineatus*	KC 757645
	Cephalopoda	*Nautilus macromphalus*	NC 007980
		*Octopus vulgaris*	NC 006353
	Gastropoda	*Cymatium parthenopeum*	NC 013247
		*Haliotis rubra*	NC 005940
		*Tricula hortensis*	NC 013833
	Monoplacophora	*Laevipilina antarctica*	KY 244020
		*Laevipilina hyalina*	KY 284344
		*Vema ewingi*	KY 244019
	Polyplacophora	*Katharina tunicata*	NC 001636
		*Sypharochiton pelliserpentis*	NC 024174
		*Sypharochiton sinclairi*	NC 024173
	Scaphopoda	*Graptacme eborea*	NC 006162
		*Siphonodentalium lobatum*	NC 005840

MITOS detected genes *atp6, cob, cox3, nad3*, and *nad4* in the *L. hyalina* consensus sequence divided in two parts, *nad2* in three parts. The parts of *atp6*, *cob*, and *cox3* are overlapping (*atp6*, *cob*) or are at least adjacent (*cox3*) and therefore were combined manually; *nad2*, *nad3*, and *nad4* do actually have non-annotated nucleotides in reverse order between the annotated gene parts. These non-annotated parts turned out to be reverse complement parts and were corrected by hand in Geneious version 6.1.7.

Annotated gene arrangements of all three monoplacophoran species were compared to each other and to other molluscan taxa (*Katharina tunicata* (NC_001636 [35]), *Sypharochiton* spp. (KJ_534306, KJ_534307 [49]), *Nautilus macromphalus* (NC_007980 [51]), *Octopus vulgaris* (NC_006353 [61]) by eye. Furthermore we searched for sequence motifs that occur in more than one monoplacophoran species with MEME Suite version 4.9.1 via the MEME web server [79].

### Phylogenetic analyses

Newly generated data for *Vema ewingi* and *Laevipilina antarctica* and reannotated mt data of *Solemya velum* [48], *Scutopus ventrolineatus* [47], *Sypharochiton pelliserpentis* and *S. sinclairi* [49] were added to a taxon-subset of the 13 mitogenomic protein coding genes (PCGs) from Stöger and Schrödl [44], comprising 18 molluscan and three lophotrochozoan outgroup taxa (Table 5). Due to visibly exceptionally long branches and unusual attraction of outgroup taxa to ingroups in previous studies [34, 44, 46, 80] and in own pre-analyses, we excluded all outgroup taxa except *Platynereis* (Annelida) that showed a short branch in pre-analyses with more outgroup taxa, and excluded all bivalve taxa but the basal protobranch *Solemya* [81]. To reduce potential long-branch attraction artifacts that are already known from previous studies (e.g., [47]), we removed the two scaphopod taxa *Graptacme* and *Siphonodentalium*. Moreover, we also ran analyses based on nucleotide (nuc) and amino acid (aa) datasets of all taxon sets without any outgroups. All single nucleotide PCG sets were translated into amino acids using the invertebrate mitochondrial genetic code. Single nucleotide and amino acid datasets of PCGs were aligned using MAFFT version 7.017 [82] implemented in Geneious under the E-INS-i algorithm with a gap open penalty of 3. In pre-analyses we masked all single gene-alignments (nuc and aa) with Aliscore version of 5th February 2008 [83, 84] by running 10.000.000.000 replicates. Hypervariable positions were trimmed with Alicut version 2.0 [83, 84]. Moreover, we ran pre-analyses where we eliminated poorly aligned and hypervariable regions of all aa single alignments via Gblocks [85] since this program is more restrictive than Aliscore. In Gblocks we applied default options except for the *atp8* alignment because this dataset would have been subsequently eliminated completely and we wanted to include the complete set of protein-coding genes; for *atp8* alignments we chose all options using a less stringent selection. The Gblocks masked single alignments were tested for best fitting evolutionary models with ProtTest version 2.4 [86] by choosing from those models that are available in RAxML (DAYHOFF, DCMUT, JTT, MTREV, WAG, RTREV, CPREV, VT, BLOSUM62, and MTMAM). We further tried to improve the aa single alignments by refining the MAFFT-alignment via Muscle version 3.8 [87]. The resulting nucleotide and amino acid individual PCG-gene alignments under the different treatments were concatenated in Geneious with the following order: *atp6, atp8, cob, cox1, cox2, cox3, nad1, nad2, nad3, nad4, nad4L, nad5, nad6*. All *atp8* alignments produced herein are missing the sequence for *Mytilus*, since this taxon lacks the *atp8* gene [43]. All Maximum Likelihood (ML) analyses were performed with the program RAxML-HPC [88], executing 500 bootstrap replicates under the CAT approximation for rate heterogeneity and the GTR model. Masking with Aliscore or Gblocks or no masking procedure as well as partitioning the concatenated dataset or not did not make any difference in the resulting tree topology and will not be discussed below. Further analyses of selected concatenated alignments were carried out with the program SplitsTree version 4 [89] to test for potential conflicts of the data.

For the two preferred datasets (aa-1, aa-2) we carried out additional analyses with Phylobayes MPI on the CIPRES Science Gateway (https://cushion3.sdsc.edu/portal2/) using the CAT-GTR model and running 4 chains for each of the datasets. Analysis of datset aa-1 was executed for 79.839, respectively aa-2 for 105.593 generations until stationarity was reached. Burn-in was set to 2000 for each of the chains. Maxdiff for aa-1 was 0,07, for aa-2 it was 0,1.

Competing phylogenetic hypotheses run on the two main datasets aa-1 and aa-2 were evaluated using the Shimodaira Hasegawa test [90] and the Approximately Unbiased test [91] in RAxML 8.2.4 [92] and Consel [90]. The PROTGAMMAGTR model was used for these analyses.

Since the phylum Mollusca diverged in the Cambrian or earlier, non-phylogenetic signal in the molecular datasets could lead to anomalous topologies due to compositional biases, substitution saturation or increased substitution rates [93, 94]. Therefore we tested our preferred single gene alignments (amino acid only) for saturation and rate heterogeneity with the programs TreSpEx [95] and BaCoCa [96].

## Additional files

Additional file 1: Figure S1. Bayesian Inference tree based on the large amino acid dataset. The tree was inferred with Phylobayes running four chains and 79.839 generations until stationarity was reached. *Loxocorone* was used to root the tree. (PDF 2 kb)
Additional file 2: Figure S2. Bayesian Inference tree based on the amino acid dataset without the two outgroup taxa *Lineus* and *Loxocorone* and the reduction of bivalve taxa to the protobranch taxon *Solemya.* The tree was inferred with Phylobayes running four chains and 105.593 generations until stationarity was reached. *Platynereis* was used to root the tree. (PDF 2 kb)

## Abbreviations

aa: Amino acid
*atp6*-*8*: ATP synthase subunits 6–8
bp: Base pair
*cob*: Cytochrome oxidase b
*cox1-3*: Cytochrome c oxidase subunits 1–3
Gbp: Gigabase pair
ML: Maximum likelihood
mt: mitochondrial
*nad1-6*: *4L*, NADH dehydrogenase subunits 1–6, 4L
NCR: Non-coding region
nuc: Nucleotide
PCG: Protein-coding gene
PCR: Polymerase chain reaction
PE: Paired-end
rRNA: Ribosomal RNA
*rrnL*: Large ribosomal RNA
*rrnS*: Small ribosomal RNA
tRNA: Transfer RNA
*trnX*: Transfer RNA for amino acid X (denoted by the one-letter IUPAC code)
