# Supplementary figures and images for: Monoplacophoran mitochondrial genomes: convergent gene arrangements and little phylogenetic signal
Source: BMC Evol Biol. 2016 Dec 16;16:274. doi: 10.1186/s12862-016-0829-3 (PMC5162086; doi:10.1186/s12862-016-0829-3)

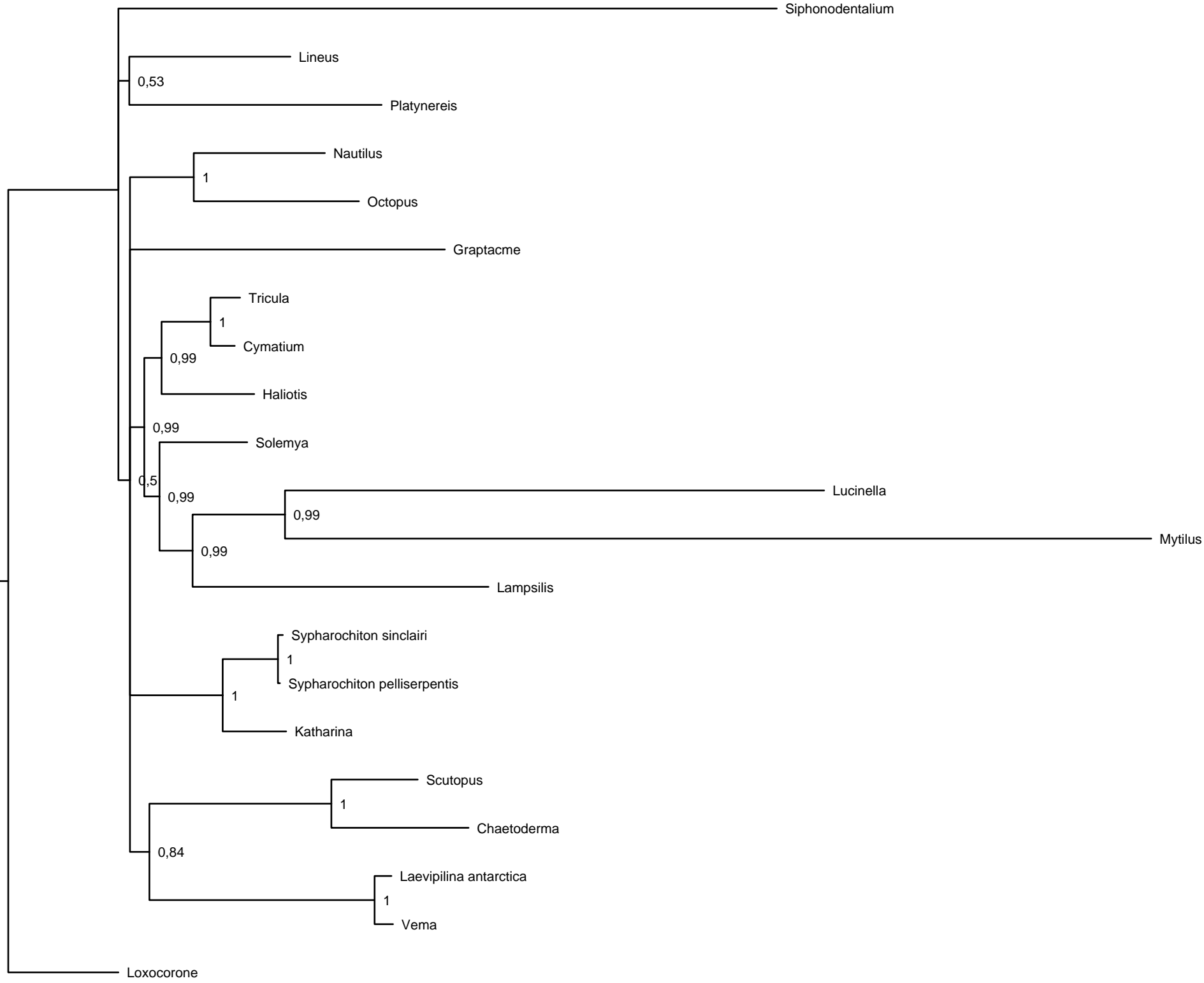

2.0

Supplement: Additional file 1: Figure S1. — Bayesian Inference tree based on the large amino acid dataset. The tree was inferred with Phylobayes running four chains and 79.839 generations until stationarity was reached. Loxocorone was used to root the tree. (PDF 2 kb) [file 12862_2016_829_MOESM1_ESM.pdf]

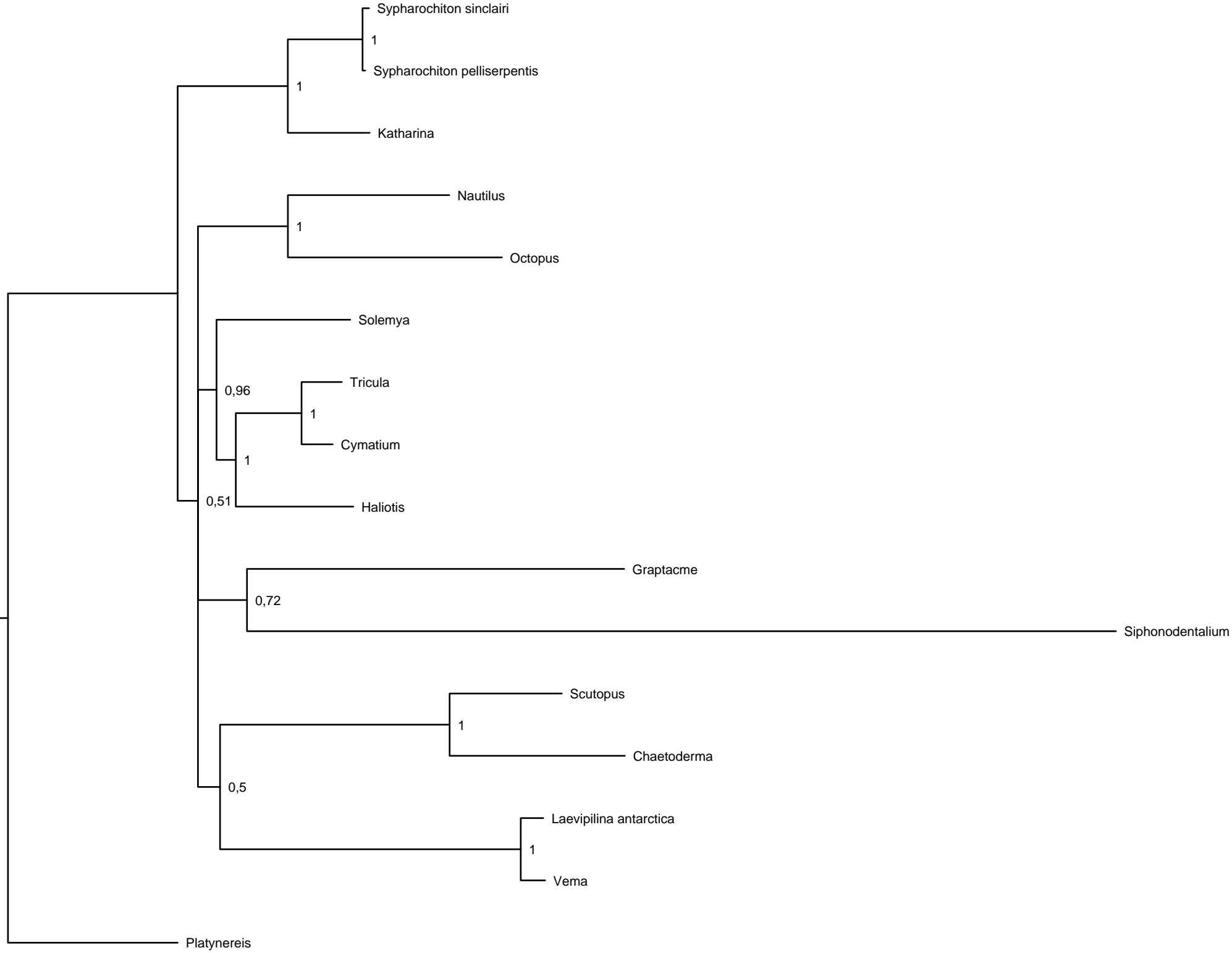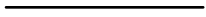

1.0

Supplement: Additional file 2: Figure S2. — Bayesian Inference tree based on the amino acid dataset without the two outgroup taxa Lineus and Loxocorone and the reduction of bivalve taxa to the protobranch taxon Solemya. The tree was inferred with Phylobayes running four chains and 105.593 generations until stationarity was reached. Platynereis was used to root the tree. (PDF 2 kb) [file 12862_2016_829_MOESM2_ESM.pdf]
